# Mutations in SERPINF1 Cause Osteogenesis Imperfecta Type VI

**DOI:** 10.1002/jbmr.487

**Published:** 2011-11-21

**Authors:** Erica P Homan, Frank Rauch, Ingo Grafe, Caressa Lietman, Jennifer A Doll, Brian Dawson, Terry Bertin, Dobrawa Napierala, Roy Morello, Richard Gibbs, Lisa White, Rika Miki, Daniel H Cohn, Susan Crawford, Rose Travers, Francis H Glorieux, Brendan Lee

**Affiliations:** 1 Department of Molecular and Human Genetics, Baylor College of Medicine, Houston, TX, USA; 2 Genetics Unit, Shriners Hospital for Children and McGill University, Montréal, Québec, Canada; 3 Department of Surgery, NorthShore University Health System Research Institute, Evanston, IL, USA; 4 Department of Physiology and Biophysics, University of Arkansas for Medical Sciences, Little Rock, AR, USA; 5 Department of Molecular, Cell and Developmental Biology, University of California, Los Angeles, Los Angeles, CA, USA; 6 Department of Orthopaedic Surgery, University of California, Los Angeles, Los Angeles, CA, USA; 7 International Skeletal Dysplasia Registry, Cedars Sinai Medical Center, Los Angeles, CA, USA; 8 Howard Hughes Medical Institute, Houston, TX, USA

**Keywords:** BRITTLE BONE DISEASE, COLLAGEN TYPE I, FRACTURE, MATRIX PROTEINS, PIGMENT EPITHELIUM-DERIVED FACTOR

## Abstract

Osteogenesis imperfecta (OI) is a spectrum of genetic disorders characterized by bone fragility. It is caused by dominant mutations affecting the synthesis and/or structure of type I procollagen or by recessively inherited mutations in genes responsible for the posttranslational processing/trafficking of type I procollagen. Recessive OI type VI is unique among OI types in that it is characterized by an increased amount of unmineralized osteoid, thereby suggesting a distinct disease mechanism. In a large consanguineous family with OI type VI, we performed homozygosity mapping and next-generation sequencing of the candidate gene region to isolate and identify the causative gene. We describe loss of function mutations in serpin peptidase inhibitor, clade F, member 1 (*SERPINF 1*) in two affected members of this family and in an additional unrelated patient with OI type VI. *SERPINF1* encodes pigment epithelium-derived factor. Hence, loss of pigment epithelium-derived factor function constitutes a novel mechanism for OI and shows its involvement in bone mineralization. © 2011 American Society for Bone and Mineral Research

## Introduction

Osteogenesis imperfecta (OI [MIM #166200, #166210, #259420, #166220, %610967, #610968, #610682, #610915, #259440, #610854]) is a heritable disease of the extracellular matrix of bone characterized by low bone mass and frequent fractures.^1^ Approximately 90% of patients carry dominant mutations that negatively affect the quantity, quality, or structural integrity of type I procollagen (MIM *120150, *120160).^2^ Type I procollagen is synthesized as a heterotrimer comprised of two alpha 1(I) chains and one alpha 2(I) chain.^3^ These alpha chains are synthesized in the rough endoplasmic reticulum, where they associate at their carboxyl-termini and assemble to form a triple helix.^3^ The posttranslational modification of residues within the triple helix, mainly 4-prolyl hydroxylation, serves to stabilize the collagen molecule.^4^

In contrast to the dominant forms of OI caused by mutations in the type I procollagen genes, in some families the disease exhibits a recessive inheritance pattern. The genetic etiology of this class of recessive OI was first described by Morello and colleagues^5^ with the identification of mutations in the gene encoding cartilage-associated protein (CRTAP [MIM *605497]). CRTAP associates in a complex with prolyl 3-hydroxylase 1 and cyclophilin B and this complex functions to both chaperone type I collagen and 3-hydroxylate a single proline residue, Pro986, in the alpha 1(I) chain.^4^ Mutations in the genes encoding any of the three complex members have since been shown to cause recessive OI (MIM *610339, *123841). 5-9 Additionally, defects in another procollagen chaperoning complex consisting of FK506 binding protein 10 (FKBP10 [MIM *607063]) and heat shock protein 47 ([MIM *600943]) have been identified as causative of recessive OI. 10-12 Together, these data identify a second mechanism for the pathogenesis of OI, that of altering posttranslational collagen processing and/or trafficking. However, other cases of OI are negative for mutations in these genes and are likely caused by other unidentified mechanisms.

Our report focuses on recessive OI type VI (MIM #610968), which was identified as a separate disease entity about a decade ago (Supplemental Table 1 summarizes key differences between classical OI [OI types I-IV], OI type VI, and recessive OI type III).^13^, ^14^ In one large series of severely affected OI patients, OI type VI constituted approximately 4% of cases.^1^ Patients with a diagnosis of OI type VI appear to be healthy at birth and do not have fractures until after 6 months of age.^13^, ^14^ This is in contrast to patients with other recessive forms of OI who typically have deformities and fractures at birth.^13^, ^14^ The pathognomonic histological finding that distinguishes OI type VI from other forms of OI is the large amount of unmineralized osteoid and blurred tetracycline labels, reminiscent of osteomalacia, despite normal vitamin D levels and normal calcium and phosphorus serum levels, coupled to the disorganization of the bone matrix, where the lamellar pattern is replaced by a fish scale appearance.^14^ Patients with OI type VI do not appear to respond to bisphosphonate treatment as well as patients with classical type I collagen defects.^13^ Thus, both the clinical and the histological findings of OI type VI suggest a unique mechanism of pathogenesis.

## Case Reports

**Fig 1:**
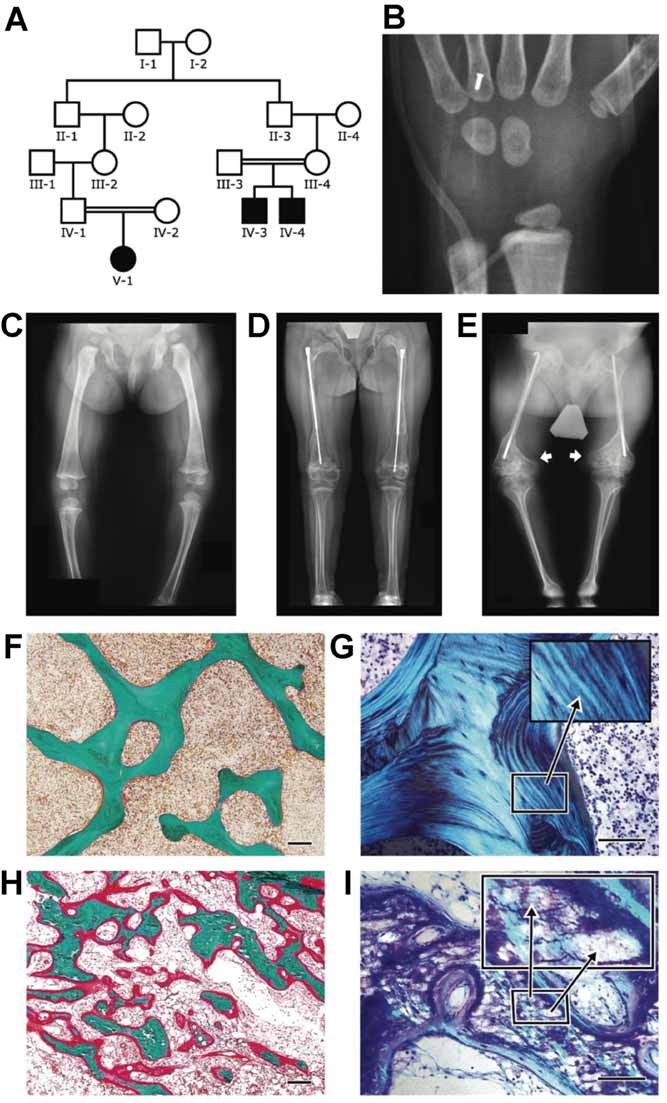
(*A*) The pedigree of the French Canadian index family. The great-grandfather of patient V-1 and the grandfather of patients IV-3 and IV-4 are brothers. In addition, subjects III-3 and IV-2 are also related (not shown). (*B*) There were no signs of rickets in patient V-1 at the age of 25 months even though bone histology showed impaired mineralization of bone tissue at the same time. The sclerotic area below the radial growth plate is a typical sign of pamidronate treatment. Lower extremity radiographs of patient V-1 at the ages of (*C*) 9 months and (*D*) 8 years, and (*E*) of patient IV-3 at the age of 21 years, showing the evolution of OI type VI from bones with normal appearance in the first year of life to bulbous ‘popcorn’ epiphyses (*arrows*) and thin diaphysis later. (*C*) A fracture crack was present in the proximal femur; however, it is too faint to be visible in the reproduced figure. (*F*-*I*) Iliac bone histology (*F* and *G*) in an age-matched control and (*H* and *I*) in patient IV-3 at 13 years of age. The left panels (*F* and *H*) show Goldner-stained sections and show a large quantity of unmineralized osteoid (*red color*) (*H*) in the trabecular bone of patient IV-3, indicative of a mineralization defect. The right panels (*G* and *I*) represent toluidine blue-stained sections of the same samples seen under polarized light, showing smooth bone lamellation (*G*, *inset box*) in the control and ‘fish-scale’ pattern (*I*, *inset box*) in patient IV-3. The quantitative histomorphometric results in patient IV-3, including dynamic measures based on tetracycline labeling, had been included in an earlier report.^13^ The size bars represent 100µm.

Patient V-1 (Fig. 1*A*) is an 8-year-old girl (part of a previously described large French Canadian consanguineous family^13^, ^14^) born at 41 weeks of gestation by spontaneous vaginal delivery and weighing 3320g (50th percentile). No limb deformities or other abnormalities were noted at birth and sclerae were white. She was able to sit independently at 6 months of age and had normal-appearing teeth. Her first fracture was a nondisplaced fracture in the proximal right femur at the age of 9 months. A skeletal survey revealed three vertebral compression fractures. The areal bone mineral density *Z*-score for lumbar vertebrae L1 to L4 on dual-energy X-ray absorptiometry was −0.1 at age 9 months. After pamidronate treatment was started at 9 months of age, no new vertebral compression fractures were observed. There were no radiographic signs of rickets at any time (Fig. 1*B*). However, she sustained 35 long-bone fractures, and underwent bilateral femoral intramedullary rodding (Fig. 1*C*, *D*). Iliac bone biopsy (at age 25 months) showed large amounts of unmineralized osteoid in bone. Biochemical parameters of mineral metabolism (serum calcium, phosphorus, 25-OH vitamin D, and parathyroid hormone), except for elevated alkaline phosphatase levels, were within normal limits at the time of biopsy and at all of the subsequent 6-monthly control examinations. The girl began walking independently at age 18 months but stopped ambulating at age 5 years because of frequent lower-extremity fractures. Growth was slow but height remained within the normal reference range (10th percentile at 8 years).

Patient IV-3 (Fig. 1*A*) is a 26-year-old man born at 40 weeks of gestation by spontaneous vaginal delivery, weighing 3520g (50th percentile). At birth, he had white sclera, normal facial features, no fractures, no limb deformities, and no joint hyperlaxity. He was able to walk independently at age 18 months, but beginning at age 6 years required a wheelchair because of recurrent fractures. His growth was severely restricted. At the age of 13 years, his height was equivalent to an average 3.5-year-old and areal bone mineral density at the lumbar spine was below that of an average newborn. He had severe scoliosis (72 degrees), bilateral coxa vara, acetabular protrusion, severe bowing of all long bones, and ‘popcorn epiphyses.’ Iliac bone biopsy showed a large quantity of unmineralized osteoid as well as absence of normal bone lamellation and evidence of a ‘fish-scale’ bone pattern (Fig. 1*F*-*I*). Serum calcium, phosphorus, and parathyroid hormone levels were within normal limits, but serum 25-OH vitamin D was slightly low (41 nmol/L; recommended range: 50-130 nmol/L) and alkaline phosphatase activity was slightly elevated (436U/L, normal: <300U/L). The urinary ratio between collagen type I N-telopeptide and creatinine, a marker of bone resorption, was elevated (three times the age- and sex-specific average value). Pamidronate treatment began at 13.7 years of age. Final height at 18 years of age was 110cm. Lower extremity X-rays at the age of 21 years showed significant widening of the metaphyses and epiphyses (Fig. 1*E*). He continued to have on average one long-bone fracture per year. Patient IV-3 has an affected brother (patient IV-4) who shares similar clinical features, and has been previously described.^14^

The third patient in this series is a 14-year-old Italian boy. He was born at 41 weeks gestation by spontaneous vaginal delivery, weighing 2950g (<10th percentile). He had faintly blue sclera, no apparent fractures, and neither joint hyperlaxity nor skin hyperelasticity. The patient's parents are not consanguineous and an older brother is unaffected. The first fracture (humerus) at age 10 months was followed by fractures of femur and forearm. Pamidronate therapy began at age 2.3 years, but his growth remained very slow and he sustained multiple atraumatic fractures. Surgical corrections of long-bone deformities began at age 9 years. Severe kyphoscoliosis was treated by spinal fusion surgery at age 14 years. He was never able to stand or walk and is dependent upon an electric wheelchair for mobility. Serum calcium, phosphorus, parathyroid hormone, and 25-OH vitamin D levels were normal and urinary collagen type I N-telopeptide levels were in the upper part of the reference range. Iliac bone histology at age 10 years showed absence of normal spongiosa architecture and osteoid excess. Analysis of collagen type I protein from skin fibroblasts did not reveal any pathological findings. Sequencing of *COL1A1*, *COL1A2*, and *CRTAP* in genomic DNA revealed no mutations.

## Subjects and Methods

### Human subjects

This protocol was approved by the institutional review board for human subjects research at Baylor College of Medicine and at McGill University. We collected blood, fibroblasts, and tissue from affected individuals and prepared DNA by standard protocols.

### Homozygosity mapping and next-generation sequencing

We genotyped DNA extracted from whole blood using GeneChip Human Mapping NspI 250K arrays (Affymetrix, Santa Clara, CA, USA), per the manufacturer's recommendations. We determined genotypes using GeneChip DNA analysis software (version 2.0; Affymetrix); analyzed using IBD Finder (Leeds Institute of Molecular Medicine, University of Leeds, Leeds, UK; http://dna.leeds.ac.uk/ibdfinder).

We subjected genomic DNA from patient V-1 to liquid capture, followed by next-generation sequencing. A full description is provided in the Supporting Information.

### Genetic analysis of *SERPINF1*

We amplified the eight exons of serpin peptidase inhibitor, clade F, member 1 (*SERPINF1*) from genomic DNA by PCR and analyzed them by dye-terminator sequencing (Agencourt Bioscience Services, Danvers, MA, USA). We analyzed the results using Sequencher 4.8 software (Gene Codes Corporation, Ann Arbor, MI, USA). Patient sequences were referenced to the Ensembl gene sequence ENSG00000132386 (*SERPINF1*). Previously known single-nucleotide polymorphisms (SNPs) (identified by the dbSNP reference database; http://www.ncbi.nlm.nih.gov/projects/SNP) were removed before analysis.

### RNA isolation and qRT-PCR

We extracted RNA from patient fibroblasts with TRIzol (Invitrogen, Carlsbad, CA, USA). We used total RNA (1µg) for synthesis of first-strand cDNA with Superscript III RT (Invitrogen). We performed qRT-PCR according to the manufacturer's protocol using gene-specific primers and a FastStart DNA Master SYBR Green I reagent using a LightCycler (Roche Diagnostics, Mannheim, Germany). Results were normalized to β-actin.

### Pigment epithelium-derived factor serum assay

We measured pigment epithelium-derived factor (PEDF) levels in human OI patient samples by ELISA, using a research kit available from BioProductsMD (Middletown, MD, USA). Briefly, we analyzed samples using a urea pretreatment step, with a final dilution of 1:10,000 and performed the assay per the manufacturer's instructions.

## Results

**Fig 2:**
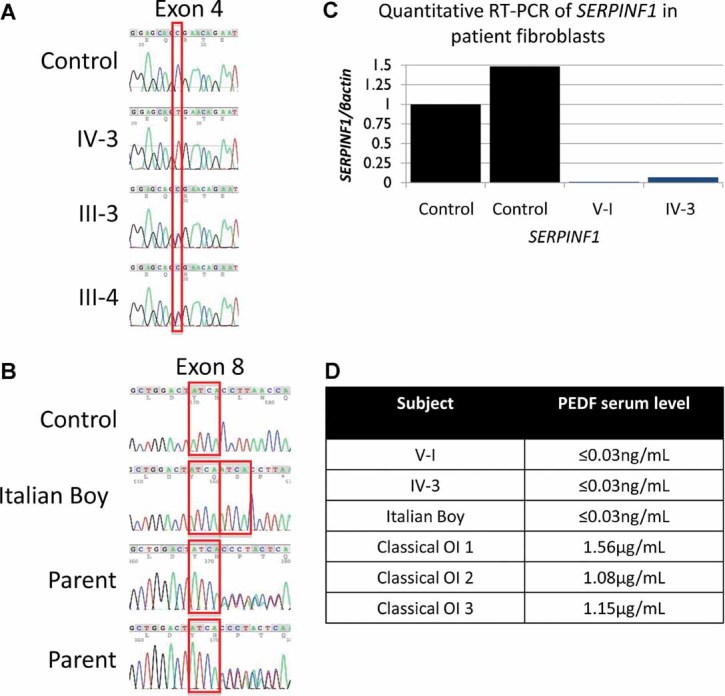
(*A*) Sanger sequencing confirmation of g.4130C>T, p.R99X in patient IV-3 as compared with control and parent (III-3, III-4). (*B*) Sanger sequencing confirmation of g.10440_10443dupATCA, p.H389f3sX392 in the Italian boy as compared with control and parent. (*C*) Quantitative RT-PCR of *SERPINF1* in patient fibroblasts shows dramatically decreased levels of *SERPINF1* transcript in patients V-I and IV-3 (g.4130C>T, p.R99X) as compared with control fibroblasts, suggesting nonsense-mediated decay of the transcript. (*D*) PEDF serum levels in all three OI type VI patients are below the detectable limits of the assay (0.03ng/mL) as compared with classical OI controls.

To identify the gene region responsible for OI type VI, we carried out homozygosity mapping using the genomic DNA of three members of the consanguineous French Canadian family (subjects V-1, IV-3, and IV-4; Fig. 1*A*). Using a minimum homozygosity size of 3 megabases (Mb) and allowing an error of 1 per 100 SNPs, a single region of homozygosity was shown to be shared by all three of these patients, at an interval of approximately 4.1 Mb, spanning from chromosome 17p13.3 to 17p13.2 and bounded by the markers at loci rs8074026 and rs1362761. This homozygous region contains 98 unique RefSeq genes. Next-generation sequencing of patient V-1 identified homozygosity for a stop mutation in exon 4 (g.4130C>T, p.R99X) in *SERPINF1* (Supporting Information) and Sanger sequencing confirmed the same stop mutation in the related patient IV-3 (Fig. 2*A*). The mutant transcript was predicted to undergo nonsense-mediated decay (NMD), resulting in a complete loss of function allele.

Sequence analysis of all *SERPINF1* exons in genomic DNA of the Italian boy identified homozygosity for a 4-bp duplication in exon 8 (g.10440_10443dupATCA, p.H389fsX392) (Fig. 2*B*). In all cases, parents were confirmed to be heterozygous carriers (Fig. 2*A*, *B*).

Because the nonsense mutation identified in patients V-I and IV-3 is located more than 50 bp before the actual stop codon, it is predicted to undergo NMD. To test this hypothesis, we assessed *SERPINF1* mRNA levels by quantitative RT-PCR. The *SERPINF1* transcript levels in patient fibroblasts (containing the p.R99X stop mutation) were reduced to less than 6% of controls, suggesting that the transcript undergoes NMD (Fig. 2*C*).

*SERPINF1* encodes the 50-kDa protein PEDF. Serum PEDF levels were undetectable in three OI type VI patients (Fig. 2*D*), but were normal in three OI patients with mutations affecting collagen type I when compared with the published normal range of 3.2µg/mL±2.0 (Fig. 2*D*).^15^

## Discussion

It has been previously suggested that mutations in the chaperone protein *FKBP10* could be responsible for OI type VI (MIM #610968); this was based upon a focal abnormal lamellar pattern that was observed in a single bone biopsy sample from a severe OI patient carrying a mutation in this gene.^12^ However, no mutations in *FKBP10* were identified in our patient cohort that was classified based on the diagnostic requirement for OI type VI of having an excess of unmineralized osteoid tissue, indicative of a mineralization defect in addition to a fish-scale pattern in their bone matrix. To the best of our knowledge, the reported patients with *FKBP10* mutations do not have the distinct pathognomonic histological features of OI type VI and instead, have a diagnosis of severe OI.^14^

In our report, next-generation sequencing of the homozygous region from a large consanguineous family with OI type VI identified a stop mutation in *SERPINF1* in patient V-1, and Sanger sequencing of this candidate gene revealed the same stop mutation in the related patient IV-3. We also identified a homozygous 4-bp duplication in an unrelated OI type VI patient. Heterozygosity was confirmed in the parents of all three described cases. At the functional level, qRT-PCR suggests that the mutant transcript undergoes NMD, which is supported by undetectable PEDF levels in the patients' serum. Recently, Becker and colleagues^16^ reported *SERPINF1* mutations in patients diagnosed with severe OI type III, but bone biopsies were not available in these cases and therefore it is not known whether these patients had the bone histological features of OI type VI. Our work suggests that OI type VI is the specific clinical consequence of PEDF loss of function.

PEDF was originally isolated from the conditioned medium of cultured primary human fetal retinal pigment epithelial cells and functions as both an antiangiogenic factor and a neurotrophic and cell differentiation factor.^17^ In bone, it has also been reported to upregulate osteoprotegerin, which inhibits osteoclast maturation by blocking RANKL-mediated osteoclast precursor proliferation and differentiation.^18^ Because our patients have loss of function mutations in *SERPINF1*, lower osteoprotegerin levels might result in increased numbers of osteoclasts. In accordance with this hypothesis, there is some evidence of increased bone resorption in these patients.^14^ If loss of PEDF resulted only in an increase in the number of mature osteoclasts, one would expect bisphosphonate therapy to be beneficial in disease management.^19^ However, OI type VI patients do not appear to respond as well as other OI patients to bisphosphonate therapy, suggesting that PEDF may have additional functions in maintaining bone homeostasis, specifically in the regulation of osteoid mineralization.^13^

PEDF has been shown to inhibit the downstream actions of vascular endothelial growth factor (VEGF), a protein expressed by chondrocytes during endochondral bone formation.^20^ VEGF stimulates blood vessel formation and allows the migration of osteoblasts and osteoclasts to the sites of bone deposition.^17^ However, PEDF is also secreted by osteoblasts and to a lesser extent, osteoclasts.^17^ Associating with type I collagen in the extracellular matrix, it may serve as a potent angiogenesis inhibitor.^21^ PEDF binds to type I collagen near the α1β2 and the α1β1 integrin binding site, suggesting that PEDF could alter integrin-collagen interactions, which have been shown to play a role in cell adhesion and, importantly, angiogenesis.^21^

Because PEDF is a potent antiangiogenic factor, it represents a promising tumor suppressor agent, and the metastases of many tumor types in mouse models are inhibited by infusion of recombinant PEDF. 22-24 Given that recombinant PEDF is available and that OI type VI patients respond poorly to bisphosphonates, systemic infusion of PEDF might constitute a viable therapeutic approach for OI type VI. At the same time, modulation of PEDF in the context of cancer and/or angiogenesis may have off target effects in the skeleton and should be addressed in future clinical trials. Finally, our data suggest that screening for PEDF levels in serum may be useful for diagnosing OI type VI patients.

Taken together, these data identify a previously undescribed mechanism for the pathogenesis of dysregulated bone mineralization in OI and, importantly, suggest that mutations in an extracellular secreted morphogen and/or signaling protein can contribute to a heritable connective tissue disorder.

## Disclosures

All authors state that they have no conflicts of interest.
