# Supplementary material for: Mutations in SERPINF1 Cause Osteogenesis Imperfecta Type VI
Source: J Bone Miner Res. 2011 Nov 21;26:2798–803. doi: 10.1002/jbmr.487 (PMC3214246; doi:10.1002/jbmr.487)
Supplement: Supplementary file 1 [file jbmr0026-2798-SD1.doc]

**Supplemental data**

**Supplementary Table 1**

|  | **Classical OI (Types I-IV)** | **OI type VI** | **Recessive OI type III** |
| --- | --- | --- | --- |
| **Mode of inheritance** | Autosomal dominant | Autosomal recessive | Autosomal recessive |
| **Gene defect** | *COL1A1* or *COL1A2* | *SERPINF1* | *P3H1, CRTAP* |
| **Presentation at birth** | Fractures present in types II and III | Normal | Fractures present at birth |
| **Histology** | Lamellae thinner than normal controls, increased matrix mineralization | Fish-scale lamellae pattern, large amount of unmineralized osteoid | Decreased cancellous and cortical bone, bone formation rate increased, mineral apposition rate decreased, bone resorption increased |
| **Tetracycline Labeling** | Dual labeling preserved | Diffuse uptake of tetracycline labels | Dual labeling preserved |
| **Dentinogenesis Imperfecta** | Variable | No | No |
| **Sclera** | Blue, greyish, or white sclera | White sclera | White sclera |
| **Skin** | Hyperlaxity | Normal | Normal |
| **Bisphosphonate therapy** | Good response to bisphosphonate therapy | Poor response to bisphosphonate therapy | Some evidence of response |

**Supplemental Table 1 Legend**

This table summarizes key differences between classical OI (OI types I-IV) and OI type VI and recessive OI type III due to mutations in *P3H1* and *CRTAP*.

**Next Generation Sequencing and Analysis**

DNA was sheared to 200 bases using the Covaris instrument. Ends were repaired to form blunt end fragments and an "A" base added to the 3' end of the blunt phophorylated fragment with Klenow (3'-5' minus). Illumina adapters were ligated to the DNA fragments and were size-selected (range 300-500) bases and enriched by a small number of PCR cycles, the library was hybridized with a custom-made bait capture kit overnight. Baits that were hybridized to captured fragments were purified using Streptavidin beads and baits were removed by RNase treatment. Capture fragments were enriched by PCR and quantified with qPCR. Finally, the enriched library was loaded onto a flow cell of the GAIIx instrument. Run was paired end 75 base.

The sequence output was aligned, using Mosaik (version 1.0.1384) to the hg19 reference sequence containing dbSNP 131 SNP masking, from UCSC’s Golden Path. The reads were converted to the Mosaik format, aligned, and sorted using the reference sequence coordinates, and then parsed to yield a multiple sequence alignment. This file was then loaded into the Avadis NGS (Version 1.0) sequence analysis tool. The following numbers of deletions, insertions, SNPs, and MNPs were detected (Supplementary Table 2).

**Supplementary Table 2**

| **Region (hG19)** | **Chr17:642928-5777023** |
| --- | --- |
| **Read Count** | **9675842** |
| **Positive** | **4839615** |
| **Negative** | **4839615** |
| **Exact Match** | **9540173** |
| **One Error Match** | **94877** |
| **Two or More Error Match** | **40792** |
| **Structural Variation** |  |
| **Insertion** | **241** |
| **Deletion** | **182** |

| **Only Homozygous with 100 of greater reads** | **Known to DB SNP 131** | **Undescribed** | **Total** |
| --- | --- | --- | --- |
| **Total events (Unique positions with >100 reads** | **1450** | **29** | **1479** |
| **Total Predicted Consequences** | **1737** | **34** | **1771** |
| **Coding region** |  |  |  |
| **STOP GAINED** | **1** | **1** | **2** |
| **SYNONYMOUS CODING** | **51** | **2** | **53** |
| **SYNONYMOUS CODING, SPLICE SITE** | **1** | **0** | **1** |
| **NON SYNONYMOUS CODING** | **47** | **2** | **49** |
| **NON SYNONYMOUS CODING, SPLICE SITE** | **4** | **1** | **5** |
| **Intronic** |  |  |  |
| **INTRONIC** | **1158** | **17** | **1175** |
| **SPLICE SITE** | **3** | **0** | **3** |
| **Genic Regions** |  |  |  |
| **3Prime UTR** | **88** | **3** | **91** |
| **3Prime UTR, Splice Site** | **1** | **0** | **1** |
| **5Prime UTR** | **17** | **0** | **17** |
| **5Prime UTR, Splice Site** | **0** | **1** | **1** |
| **Non-Genic Regions** |  |  |  |
| **DOWNSTREAM** | **163** | **4** | **167** |
| **UPSTREAM** | **162** | **2** | **164** |
| **Miscellaneous** |  |  |  |
| **EXONIC (Coding Information Absent)** | **22** | **0** | **22** |
| **GENIC (Transcript information for gene absent)** | **19** | **1** | **20** |
